# Techno Trend Awareness and Its Attitude Towards Social Connectedness and Mitigating Factors of COVID-19

**DOI:** 10.3389/fpsyg.2021.637395

**Published:** 2021-05-25

**Authors:** Vijyendra Pandey, Neelam Misra, Rajgopal Greeshma, Arora Astha, Sundaramoorthy Jeyavel, Govindappa Lakshmana, Eslavath Rajkumar, G. Prabhu

**Affiliations:** ^1^Department of Psychology, Central University of Karnataka, Gulbarga, India; ^2^Department of Microbiology, Gulbarga University, Gulbarga, India; ^3^Department of Social Work, Central University of Karnataka, Gulbarga, India

**Keywords:** social connectedness, belongingness, technological trend awareness, COVID-19, indigenous knowledge, Indian culture

## Abstract

While COVID-19 has taken a toll on many professions and livelihood of all walks of lives, technology has amplified its intrusion to ease the necessities. Innovative technology, therefore, has improved the glitches and provided the software to adhere to these new normal. However, individuals' awareness and attitude toward the advancements of these technological trends need to be addressed. Although the government has taken measures to prevent and curb the growing cases for COVID-19 with the help of technology, the support from the individuals would depend mostly on their level of awareness and the attitude toward those measures. The present qualitative study explored the techno trend awareness, perception and attitudes of techno experts and technical professionals toward social connectedness and mitigating factors of COVID-19. Besides, it also explained individuals' shift toward virtual interaction to maintain social connections during the pandemic. The thematic analysis generated four prominent themes. Social Connectedness, emphasized on the emotional connections that created a positive feeling of belongingness. Technological Advancement provided three sub-themes highlighting perception, techno trend awareness and desirable attitudes toward the mitigation of COVID-19. The categories under Treatment and Preventive Measures indicated the enhanced self-care of individuals and also the tendencies to minimize the spread of diseases. The emergence of the theme Inclination toward Indigenous Knowledge, which is an important finding, indicated the techno-experts inclination toward the indigenous knowledge amid vague scientific shreds of evidence.

## Introduction

Generational cohorts are a group of people who experience events similarly and are loosely defined by their birth year. Generation “Z” were born between 1997 and 2015, Generation “Y” or “Millennials” were born between 1981 and 1996, while Generation “X” were born between 1965 and 1980 (Dimock, 2019). The literature has quite evidently indicated that there exists a significant positive correlation between techno trend awareness and Generation “Z”, as they were born into the digital world (Othman and Rashid, 2018). While research indicates that for “Millennials” the utilization of technology has been a device for entertainment and hedonistic needs, individuals of generation “X” engage with technology to search for information and fulfill utilitarian functions (Calvo-Porral and Pesqueira-Sanchez, 2019). Owing to rapid developments in technology, it has become a necessity for individuals to be in constant touch with technology, especially during the COVID-19 pandemic. The pandemic continues to work as a catalyst for scientists, researchers, and technology experts to invent applications that could meet the challenges and mitigate the spread of COVID-19.

Studies have indicated that the use of Artificial Intelligence (AI) increased drastically when the pandemic began. It has been well-utilized for things such as tracking down the patients who had come from other countries or states, as well as detecting fever and other symptoms through facial recognition. Drones and robots were used to deliver products or food, to disinfect public places, and to identify if people were maintaining social distancing. AI and other technological advancements were incorporated for diagnostic purposes, for predicting patient's health recovery and mortality rates, drug repurposing, and for other medical and social utilities (Kumar et al., 2020a; Vaishya et al., 2020; Zhou et al., 2020). Although these technological developments are being utilized at a fast rate to meet current pandemic need, the extent of awareness of these advanced developments, in terms of privacy, domain knowledge, and how user-friendly these technologies are, has not been so clearly discussed in the literature (Leslie, 2020).

### Technology Acceptance: Theories and Models

Technology trend awareness refers to the skill of an individual to be aware and mindful of new and popular technology that has been gaining widespread acceptance across concerned industries or markets (Rahimah et al., 2018). This may also include an individual's ability to recognize and understand the utilities as well as benefits of these technologies for a successful business (Rahimah et al., 2018) and other areas like health, which is our concern in this paper. Numerous studies have given significant importance to technology acceptance theories. For instance, the innovation diffusion theory (Rogers, 1962) stated that attributes like compatibility, relative advantage (perceived usefulness), observability, trialability, and complexity (ease of use) were the parameters that would help to determine an individual's adoption of innovation (Momani and Jamous, 2017). The theory of reasoned action developed as a theory of planned behavior (Ajzen, 1991), has been widely used in several applications in diverse fields, i.e., technology, by the addition of perceived behavioral control. The Technology Acceptance Model (Davis, 1985) has further replaced the theory of reasoned action with two additional factors: *perceived ease of use* and *perceived usefulness*. Model of *PC* Utilization *(MPCU)* gives a better understanding of the influence of several factors such as social norms, individual habits, perceived outcomes of usage, facilitating conditions, and direct as well as indirect experiences of computer usage on individuals' behavior (Thompson and Higgins, 1991).

Motivational Model (MM), which was proposed by Davis et al. (1992), highlighted the application of extrinsic and intrinsic motivation to understand the use and adoption of technology. This was recognized due to perceived usefulness as the extrinsic motivator and perceived enjoyment as an intrinsic motivator. Furthermore, a Unified Theory of Acceptance and Use of Technology (UTAUT) proposed by Venkatesh et al. (2003) indicated that effort expectancy, performance expectancy, and social influence proved to be the factors that determine the intention and behavior of technology use. Studies focusing on Information and Communication Technology (ICT) have been incorporating recent developments of deterring factors in their theories to fulfill needs and improve facilities, as these are essential prerequisites for the technology's utilization, realization, and acceptance (Momani and Jamous, 2017). Although ICT creates innovation and incorporates the theories into action, a lack of knowledge and proper awareness would hinder them from availing the benefits of these services provided by the government and private sectors (Reffat, 2003). The current study, therefore, aims to explore and understand the attitude, perception, and awareness of technological experts and skilled individuals toward these technological innovations to sustain life and life activities during the pandemic.

### Awareness: Self, Other, Group Perspectives

Any individual's awareness of any event depends on various components. According to Greenspan's model 1981, an individual's social awareness is dependent on three main factors: *sensitivity* (perceptual component), *insight* (interpretation component), and *communication* (Black and Rojewski, 1998). When an individual is *sensitive*, he or she will be able to recognize the happenings in the surrounding or a social event and would be able to identify others' experiences at such events. The second component, *insight*, indicates the ability to understand the reasons (why it happened) behind the situations and to comprehend possible factors for others' behaviors in those situations, as well as to exhibit social reciprocity. Depending on these two factors, the third component, *communication*, reflects on the ability to take action in a given situation (Black and Rojewski, 1998). In addition to this model, Polanyi (1969) and Wegner (1982) have tried to build on the idea of focal and tacit awareness that human beings experience. At first, usually, individuals would focally become aware of a situation by constituting the surroundings, comprehending the event, and then evaluating the event.

A social event or social context influences an individual's behavior and therefore forms a relevant factor to determine human interactions (Rakotonirainy et al., 2009). According to Dey (2001), when individuals interact, they use all their sensations to listen and watch attentively and try to perceive, think, and behave following others' intentions. People adapt and react to situations and conversations based on the interacting partners as well as on their intentions. Social meanings and contextual references are generated through such widened interactions, giving rise to social norms. Thus, social interactions are necessary for social norms and human group behaviors (Rakotonirainy et al., 2009). Furthermore, emotional awareness facilitates positive interactions, leading to improved social well-being and social support (Beaman, 2008). However, awareness of oneself and others in the surroundings and awareness of the situation becomes an indispensable part of human interactions (Rakotonirainy et al., 2009).

The main objective of the study is to explore techno trend awareness and practices of techno experts to mitigate and reduce the psychological and physical ailments caused by the COVID-19 pandemic and associated protocols.

### Social Connectedness: Belongingness Hypothesis and Shared Stress

The information of awareness means being aware of others' whereabouts, including others' feelings, experiences, moods, and activities. These play a major role in strengthening the bond of belongingness and relatedness. Social Connectedness could be understood by these experiences, mainly subjective, that lead to feelings of belongingness and relatedness that are dependent on the salient relationships and social appraisals (Van Bel et al., 2009). The belongingness hypothesis explains that individuals have a dire need to form and maintain interpersonal relationships at least to a minimum quantity of positive, lasting, and significant relationships (Baumeister and Leary, 1995). Classical experimental research highlighted the ways where groups' socialization and intergroup relations are maintained using common co-operative goals and establishment of behavioral and emotional patterns of communications, leading to feelings of cohesiveness (Tajfel and Billig, 1974; Brewer, 1979; Sherif et al., 1988). Thus, it is not only through positive situations that individuals bond, but individuals can also connect more strongly or positively when they share similar negative experiences or circumstances, which was supported by the social comparison theory (Festinger, 1954; Latané et al., 1966; Kenrick and Johnson, 1979). This could indicate that the adverse and negative circumstances shared by everyone due to the COVID-19 pandemic could bring people closer. Therefore, one can assume that under such adverse and negative situations, the emotional and social interactions between individuals would be strengthened and the bond would be stronger. This study intends to explore this line of thought to understand the social connectedness under such shared stressful circumstances.

### Technology: COVID-19 and Mitigating Factors

Artificial Intelligence, Information and Communication Technology, and other innovative technologies are being incorporated to mitigate and curb the spread of COVID-19 as well as to maintain social relationships. The use of AI to analyze, predict, and treat COVID-19 patients, as well as to prevent contamination, has been well-acknowledged (Kumar et al., 2020a), although the attitude to all these experiences needs to be explored at a deeper level. Further, AI has also been applied for accurate COVID-19 diagnosis by using neural networks (COVNet), 3-dimensional deep learning model, and by extracting radiological features. AI-incorporated models, as well as machine learning and deep neural network models, continue to contribute greatly toward predicting protein structure, generating novel drug compounds, preventing unsupervised clustering, and various other such preventive and treatment measures (Kumar et al., 2020a).

Social Connectedness was found to be critical for good and balanced physical as well as mental health, while poor connectedness correlated with poorer mental health, like depression and anxiety (Saeri et al., 2018). To curb the increasing rates of mental and physical illness during the pandemic, many online medical, counseling, and therapy sessions were organized by various universities and institutes, which were possible only because of the improved technological facilities under such circumstances. While maintaining social relationships using online social network applications might help people to stay connected even during lockdowns, measures to mitigate the spread post-lockdown have also begun. ICT's implementation of physical sensors and improving Heating, Ventilation, and Air-Cooling (HVAC) systems to maintain health and safety and achieve sustainable development goals have been in the limelight (Franco, 2020). With the emergence of more and more innovative applications, the individuals' extent of awareness, attitude, and perception toward their experiences is highly ambiguous. Additionally, since literature has proved that culture has an effect on technology acceptance, India's cultural values could contribute to the attitude formations which would thus influence their perception of techno trend facilities (Fusilier and Durlabhji, 2005; Bandyopadhyay and Fraccastoro, 2007). Culture, according to Hofstede (2001) refers to “the collective programming of the mind which distinguishes the members of one group of people from another.” Relative to other western civilizations, India has been considered as a collectivist culture which highlights the presence of high social pressure and social influence, indicating the strength of conformity values. Conformity values emphasizes on the belief system to be obedient and maintain social order as well as to cause no harm to others (Konsky et al., 2000). While conformity influences individuals' attitude toward technology use (Bandyopadhyay and Fraccastoro, 2007), tradition plays a role in the individual's belief to follow and maintain their cultural, family, and religious traditions (Schwartz, 2012). Traditions influence individuals to follow certain rituals and customs to maintain social bondage and gain social acceptance (Banerjee, 2008), which could alter and aid in attitude formation and technology acceptance. Thus, the role of their cultural and professional identity, in addition to the underlying Indian specific cultural and traditional values, might play a major role in the formation of their awareness, perception, and attitude toward the amenities provided by the techno trend facilities (Slay and Smith, 2011; Karjalainen, 2020). Therefore, the study intends to explore the techno trend awareness of techno experts toward advanced tests and preventive measures to mitigate the spread of COVID-19.

## Methods

### Design

The present research used an explorative qualitative approach to discover the techno trend awareness, perception, and attitudes of techno experts toward social connectedness and mitigating factors of COVID-19. Furthermore, it aims to understand the techno experts' shift toward virtual interaction to maintain social connectedness with close family and friends, as well as with others with whom they interact during the lockdown and the spread of this pandemic.

### Participants

The participants included professionally qualified techno-experts of their respective areas who were equipped with enhanced and updated technical skill and knowledge. It was also mandatory that they were specifically active in handling and assessing the COVID-19 related testing, diagnosis, and treatment as well as the other technical professionals who were dealing directly with people facing COVID-19-related problems. Experts who were post-graduates in science with a minimum qualification in a technical background and who had knowledge about testing of COVID-19, as well the professionals involved in treatment, were included for this study. The participants had to be aware and mindful of technology that was becoming popular in recent times, especially that which has been accepted readily in relation to COVID-19 diagnostic and preventive measures. The participants who were experts but not dealing with COVID-19 patients were excluded from the study. Additionally, those who did not agree to contribute enough time for providing the data (due to the scarcity of manpower, they were assigned more responsibilities) were excluded. Also, those who lacked a basic level of English (reading and writing) were excluded from the present study. These kinds of information have been obtained from the participants over telephonic and personal conversation. Owing to the ethical considerations, informed consent, and confidentiality of the participants, further details and their identity are not revealed here. Purposive sampling technique was used wherein the researcher acquired the consent of the participants to participate in the study. A total sample of 31 adults aged between 26 and 40 years (Mean age = 31.97, SD = ± 3.851; Males = 16; Females = 15) who showed a willingness to participate in the study were selected.

### Procedure and Data Collection

The participants were informed about the purpose of the study, and verbal consent was taken from each of them. Initially, a screening was done to establish a strong rapport through telephonic interviews and their permission and availability for participating in the study was sought. To accommodate their work schedule and to reduce lengthy conversations given their strict and overloaded responsibilities, semi-structured open-ended questions were administered through circulated Google Forms from the selected participants. The intended semi-structured open-ended questions were asked to understand their techno trend awareness, perception, and attitude toward the diagnostic and preventive measures for COVID-19. Further, they were also asked how technologically-adapted facilities were useful for increasing social connectedness and eventually in helping to mitigate the newly emerged problems related to COVID-19. The questions were asked in English to explore the opinion pertaining to major objectives of the study i.e., (a) *What is your opinion on the rapid testing and non-rapid tests designed to diagnose COVID-19?* (b) *What is your perception about home quarantine for asymptomatic patients or patients with mild symptoms?* (c) *Since the stay-at-home order began, how often have you been talking or chatting with friends or family?* (D) *Have you felt that connection through digital media was similar to that of meeting in person?*. On average, each participant took ~40–50 min to complete the questions (and received their feedback regarding the entire schedule) and a follow-up session was done to recheck and confirm their responses through telephonic conversation.

### Data Analysis

Qualitative thematic analysis was employed to analyze the data. The data obtained from Google Forms was downloaded and systematically arranged by the researcher; additionally the researchers' conversations were also used to confirm the meaning of the participants' answers to the questions. The verbatim was well-read and carefully studied by researchers to understand the underlined meanings of their responses and finalize the major themes. Graneheim and Lundman's 2004 approach was used to analyse the data. The process of abstraction was utilized to code and create categories and themes. All the words, sentences, and paragraphs that indicated similar meanings were put together and condensed. Categories with similar meaningful homogeneous data were combined. Major themes, sub-themes, and categories were formed through the process of abstraction.

## Results and Discussion

The COVID-19 pandemic has encouraged experts of all fields to help the community as well as to achieve a common goal of making individuals' lives better. Government and private sectors have implemented various policies and taken technologically supported measures to fulfill these goals. However, the success of these measures largely depends on the awareness and support of the individuals who directly use these technological facilities. Recent literature indicated that COVID-19 has led to increased mortality and increased morbidity globally; it specifically caused a greater risk of mortality on patients with comorbidities (Wang et al., 2020).

The techno experts' awareness and attitude in the analysis identified three ways– *home quarantine, early diagnosis*, and *treatment and preventive measures*– to reduce or prevent the mortality and morbidity due to COVID-19.

### Home Quarantine and Social Connectedness: Perception, Awareness, and Attitude

#### Perception and Attitude Toward Home Quarantine

The techno experts shared their perception, awareness, and attitude toward the protocol of following home quarantine, especially for asymptomatic patients and patients with mild symptoms. Most of the participants had a positive attitude toward this protocol. They advised that patients with asymptomatic and mild symptoms should follow home quarantine instead of being hospitalized due to a lack of facilities in hospitals. A male subject, PSC, responded, “*A home quarantine is best for asymptomatic patients with proper guidelines, and treatment is good instead of hospitalizing them as they don't need much attention or respiratory aid*.” Another male respondent, RR, opined that “*If the patient is less than 50 years [old] without comorbid conditions with no or mild symptoms, they can be home quarantined. It helps the actual needy patients for bed availability in hospital*.”

This indicated the concerns and support of techno experts and directions to the patients in following the precautions by themselves for their fast recovery. Song et al. (2020) have also exerted the public's supportive attitude toward home quarantine and following other necessary protocols, keeping in mind the nation's larger interest. It has also been observed that India's higher tendency to conform to rules and formal obligations that are considered important for the larger group (Bandyopadhyay and Fraccastoro, 2007) might have also impacted these behaviors. So, this study provides an insight into the possible perception people may have.

Digital media and online apps which represent the techno trends in the market to ease communications have allowed people to increase their social connectedness while being home quarantined and physically distanced from each other.

### Attitude Toward Online Interaction

The pandemic has caused people to stay connected using virtual applications. Similarly, the irritability and feelings of loneliness and boredom which were caused by home quarantine have reduced with the help of online interactions that enhanced social connectedness. Although all the respondents admitted that virtual interactions are not as good as compared to face-to-face or physical communication, it still helps social connectedness For example, a respondent, AS, said: “*Virtual interaction may be good for some days, it has brought us closer and made us feel that we are together and so helped us to be confident and we felt supported but [it] still doesn't bring the same love and affection as with Physical interaction*.”

Some respondents agreed that digital interactions have helped them to stay connected, depending on the closeness of the relationships. For instance, a participant, SSD, responded, “*Yes, digital interactions help to reduce the distance [during] such disaster times but it can never replace the physical presence. And it all depends on the kind of relationship we have with the person*.”

Overall, digital interactions have proven their major role mitigating the boredom, loneliness, and irritability caused due to home quarantine during the pandemic (Oh et al., 2014; Gabbiadini et al., 2020; Subrahmanyam et al., 2020). The participants suggested that it is the association of the quality of interaction in establishing psychological well-being and social support that needs to be emphasized irrespective of it being digital or face-to-face, indicating a greater emphasis on society's social bondage, social interdependence, and social sharing. This in turn suggests creating a collective identity which is a representation of the Indian cultural values (Banerjee, 2008). Moreover, in India relationships are a major concern. Additionally, a study by Gabbiadini et al. (2020) also emphasized the positive role of digital technologies in reducing psychological distress experienced due to social isolation, indicating toward a positive attitude toward the role of technology trends to help individuals during the pandemic.

### Online Social Reunion

The pandemic allowed individuals to reunite with old friends and acquaintances, which was possible mainly because of social media networking sites. This sub-theme emphasized the positive effects on digital media in reuniting with childhood and old friends and acquaintances.

For example, a respondent, SR, emphasized that “*Yes definitely, [a] few people [have] been connected, who remained out of sight for some time. This made me happy cause I never thought I would have time to reconnect with old friends. We all are so busy in our daily life activities*.”

A few responses indicated the time this pandemic has offered in aiding to reconnect with long lost friends which otherwise would not have been possible. Hacker et al. (2020) emphasized the ways social reunions have taken place during the pandemic, which gave rise to an increased number of interactions with families and friends.

Overall, techno experts' awareness revealed that techno trends have aided virtual interactions in a way that has helped them to reduce boredom and loneliness, increase the feeling of belongingness, and thereby enhance social connectedness even during the time of the pandemic. These examples are emphasizing the importance of technical aids and their positive attitude toward the techno trends as well as its positive effect on social connectedness which has helped in mitigating the psycho-social problems caused by COVID-19. Similar findings highlighted the role of social connectedness in reducing stress and fatigue as well as promoting resilience against COVID-19 related worries (Nitschke et al., 2020).

### Early Diagnosis: Awareness and Attitude Toward COVID-19 Diagnostic Tests

This main theme emphasizes on the techno-experts' and technical professionals' awareness, attitude, and perception toward the facilities technology has offered in mitigating COVID-19 crisis. The use of Artificial Intelligence (AI) and radiology features to diagnose COVID-19 have been covered in this main theme. Although several advanced technologies exist, the standard protocol on which technology and how that suggested technology is to be used is still not very precise and clear. This ambiguity has been mentioned by techno experts of this study in an unquoted manner wherein they opine that the ambiguity has created confusion on using the technologies based on their convenience, availability, and personal experiences, leading to varied results and interpretations of testing and treatment.

### Rapid and Non-rapid Testing

There are two main types of tests that are being widely used across the globe: Rapid antigen tests (U.S Food Drug Administration, 2020), also known as the rapid diagnostic test- RDT and RT-PCR tests–real-time reverse transcription-polymerase chain reaction. Among the various alternative diagnostic tests, RT-PCR tests have been considered as the most efficient (Júnior et al., 2020). Although sources revealed that RT-PCR tests take time to produce results, the accuracy is far better than the antigen tests, which are quick in terms of availing the results of the test (World Health Organization, 2020a). In line with this difference, this theme indicated awareness as well as the attitude of techno-experts. While some indicated the accuracy of non-rapid tests, a few recommended considering the technicians' efficiency in conducting the tests. A female expert, SSD, responded, “[The] *Rapid test just gives a superficial idea about the condition which must be correlated with clinical symptoms and other issues of patients. While non-rapid tests provide an accurate report (majority cases) but it requires well-experienced staff* ”. However, citing the immediate need, a male expert, SR, responded, “*Testing is very crucial in deciding the emergence of COVID-19 /non-COVID-19 patients. As there is no perfect vaccine for COVID-19, rapid antigen testing becomes very pivotal*.”

Overall, though the need of the hour demands rapid testing, the experts in this study opined that accurate testing is more required than rapid testing, especially since they all agreed that non-rapid tests are more reliable due to the specificity and sensitivity. However, accurate testing would mean slow and expensive testing, which is a crucial deciding factor for whether rapid or non-rapid tests are used. However, with the current confused state and with no other option, rapid testing seems to be proving beneficial, at least at a superficial state (New York Times, 2020; Radcliffe, 2020).

### Awareness and Attitude Toward BALF Tests

Bronchoalveolar Lavage Fluid tests are used for the diagnosis of lung pathology. A bronchoscope is passed into the lungs either through the nose or the mouth to recollect the fluid, which was squirted into the lung for analysis (Guthrie, 2012). Since COVID-19 majorly affects the respiratory system, studies have been proving that the BALF test could help to improve in determining the diagnosis faster. A male expert, RR, suggested “*Bronchoalveolar lavage sampling is an invasive procedure. It can't be done on all patients, only for patients who have severe disease Bal fluid can be collected*.”

Most of the responses indicate a similar line of thought, which is the efficacy in the technician's testing ability and the practical feasibility options to conduct these tests in large samples. Most responses about the BALF tests have a positive attitude. Some studies also recommended BALF tests. However, experts suggested that the testing should not rely on BALF alone, it should be integrated with other testing procedures as it cannot be used to confirm COVID-19 alone but instead helps to resolve the complex diagnostic issues (Jahromi et al., 2020; Ora et al., 2020).

### Awareness and Attitude Toward Physiological Measures

This sub-theme emerged with the significant aspects such as the data collected from heart rate, sleep patterns, deep neural networks, and X-ray scans helpful in diagnosing or detecting COVID-19.

Concerning the data related to heart rates' and sleep patterns' contribution in detecting the virus, mixed responses emerged. For example, a male expert, SR, suggested, “*Sleep is a very important factor to defeat COVID-19. As per one study, sleep produces some proteins in the body, thought to be antiviral in nature*.” However, some responses have also indicated an unclear perspective. A female respondent, SSD, emphasized that “*As the symptoms vary from person to person, with age, previous history, etc. It may require immense research and strict follow-up studies”*.

Also, concerning the correlation of X-ray results with COVID-19 diagnosis, most of the participants were not aware that X-rays could also give a significant or effective diagnosis. However, some respondents highlighted their logical reasoning. A female subject, SST, said, “*Studies have said X-Ray or CT scans have limited scope to diagnose COVID-19 as the infection seen in the images might be due to other illness also*”. Another female subject, SC, responded, “*X-ray & CT scans [are] useful to know the changes in lungs but not in all groups of COVID positive patients*.”

Overall, the participants in this study revealed that they possess a general awareness of the technology-applied tests to detect COVID-19; however, they assumed an unambiguous attitude toward the efficiency or accuracy in diagnosis. However, AI incorporation seems to be supportive in the long run for such clinical diagnosis (Kumar et al., 2020a; Ozsahin et al., 2020).

### CO-rad Efficiency

This sub-theme emerged with an indication of the participants' awareness and attitude of COVID-19 Reporting and Data Systems (CO-RAD). CO-RADS is a standardized classification and reporting system for patients with COVID-19 symptoms, developed for a range from moderate to high prevalence setting. The CT findings would indicate if the level of COVID-19 suspicion is very low (CO-RAD 1) or very high (CO-RAD 2) (Prokop et al., 2020). This sub-theme revealed the experts' awareness and attitude toward CO-RAD's feasibility to assess the severity of COVID-19 infections as well as risk factors of COVID-19 negative patients.

For instance, a male subject, RR, responded, “*To some extent, it gives us…. statistics about [the] percentage of people infected, rate of spread, incubation period, and many other variables.”*

Overall, the participants perceived that CO-RAD is a good monitoring system, but it requires the genuine and committed efforts of the staff, technicians, and media involved in reporting the data faithfully without which the entire purpose would be lost. A few studies also indicated that CO-RAD has good diagnostic accuracy and the results can be relied on, however, researchers recommend that assessments and diagnosis should never be based solely on any one kind of testing as there have been some cases that could not make the perfect differential diagnosis (Fujioka et al., 2020; Prokop et al., 2020).

### Treatment and Preventive Measures: Mixed Approach

Techno experts have indicated an overall positive attitude toward the utilization and efficiency of the technological trends or advanced products like virtual media for enhancing social connectedness and early diagnostic measures for identification and spread of the disease. However, due to the ambiguity involved in the nature of the COVID-19 variants, symptoms, prevention, and treatment protocols, the experts also indicated an ambiguous attitude toward the standard scientific treatment and preventive measures. The techno experts' identity concerning their culture and profession has greatly influenced the formation of their attitude toward these treatment and preventive protocols. Techno experts' cultural identity and traditional knowledge might have led them to incline toward their traditional and cultural practices.

Cultural identity indicates an individual's personal identity that represents one's self-understanding of a given culture or culture specific traits (Karjalainen, 2020). In this context, techno experts emphasized their trust on traditional roots, especially under the pretext of an ambiguous situation. Their cultural identity has led them to acknowledge and follow traditional and indigenous-based practices which have proven to be useful for their families or ancestors. For example, a male, AS, responded that “*In the end, our culture comes back to us*.”

However, professional identity also played a huge role while making decisions about the kind of treatment and preventive measures to be used. Professional identity revolves around their professional self-concept which is influenced by their different attributes, values, beliefs, motives, and experiences (Slay and Smith, 2011). To avoid the ambiguity toward standard protocol and traditional -cultural preventive measures brought upon by their cultural and professional identity, techno experts relied on a multifaceted approach by integrating both the scientific and traditional or indigenous knowledge to mitigate the spread of COVID-19.

Therefore, there are two main subdivisions under this mixed treatment and prevention approach: Techno trend measures and Traditional Indigenous practices.

### Techno Trend Awareness for Treatment

The theme emphasizes the use of technological advancement in the race to mitigate the spread of COVID-19.

*i) Perception of telemedicine consultation*:

This sub-theme emerged, highlighting the importance of online health or medical services during the lockdown and pandemic crisis. A female subject, SSD, said, “*It seems to be a better option as going to hospital may increase the risk of transmission to large groups*.”

Most of the respondents agreed that to contain the spread of COVID-19 it is advisable to opt for online medical services. Also, online medical services could be very helpful for asymptomatic patients as well. However, the use of telemedicine services has only improved the medical services during the COVID-19 crisis while also mitigating the spread of the virus (Monaghesh and Hajizadeh, 2020; World Health Organization, 2020b).

*ii) Awareness on Plasma Therapy*:

Scientists have been in a race to find a vaccine to tackle COVID-19 since it emerged. Although various treatment trials are ongoing, only a few vaccines are available for frontline health workers at present. It may take some time in order to reach to a larger segment of the population. Until the vaccination drive reaches more people, it is necessary to continue with symptomatic treatment as a cure. Plasma therapy has also been integrated as one of the treatment measures that might prove an effective cure for COVID-19, which emerged in the analysis. A male subject, JP, responded, “*Yes, plasma therapy is more useful for the newly infected people to fight against infection. Plasma therapy involves the use of antibodies from cured persons for newly infected people. Antibodies help boost up the immunity and cure patients as easily as possible*”. Although some experts suggested that plasma therapy could prove helpful in curing a patient, they also highlighted the possibilities of it not working.

In addition to this, most of the participants have also rated Remdesivir as the most efficient drug in combating COVID-19 based on the data received from media and other sites. Since COVID-19 is a new disease, prior research does not exist; therefore, even the participants find it difficult to logically imply that plasma therapy would work. Lack of research is another highlighted reason for this ambiguity. Literature also indicates the dearth in this regard as the myriad of studies done are still contemplating the effectiveness of plasma therapy due to ongoing clinical trials. While it proved to be effective for some clinical trials, the long-term effects are not known yet (Agarwal et al., 2020; Duan et al., 2020; Ghosh, 2020).

### Techno Trend Awareness for Prevention

*i) Attitude toward PPE kit*:

Literature has indicated that good quality Personal Protective Equipment (PPE) is required for front-line workers to serve safely and their availability ensures a major factor in combating the pandemic (Sharma et al., 2020).

A male respondent, JP, said, “*Until we habituate to it, wearing PPE kits for a longer period has many adverse effects on health as we can't eat or drink. It mainly affects homeostasis and causes heat generation in body results in extreme burning sensation of palms and also causes mouth ulcers*.”

Overall, the experts opined that, although PPE kits protect the workers from the virus, being in the kit for a long duration hampers their activities and causes other health difficulties. It is also suggested that PPE kits may adversely lead to skin infection and behavior changes among staff which could make them vulnerable to contracting the virus (Foo et al., 2006; Atzori et al., 2020; Kantor, 2020).

*ii) Attitude toward Techno Trends in reducing COVID-19 spread*:

This sub-theme emerged with an emphasis on the various technological advancements that were developed to mitigate the spread of the virus. Participants opined on the use of robotics and drones for disinfecting and food delivery systems as well as the use of the wristband alert system for contact tracing. A male respondent, SR, opined that “*Well, these things can become technically successful in developed nations; [in] a country like India, it takes a lot of toll on our economy, our government has to pour lots of money on this*.”

With concern to the uses of a wristband alert system, a male subject, RR, responded, “*It depends upon the people, how they accept [it] because many are not using mask only, I doubt they will accept and, moreover there is social stigma created*.”

Overall, the participants being techno experts themselves, this theme revealed the economic and genuine concerns about mitigating COVID-19. India is still a developing country and to use drones and robots on a wide scale requires strong finance, which the country might be lacking. Therefore, self-awareness is required more than any technological tool. However, many studies have supported the efficiency in the usage of drones and robots where it has helped and reduced direct contact, which thereby aided in mitigating the spread of COVID-19 (Euchi, 2020; Kumar et al., 2020b; Preethika et al., 2020).

*iii) Attitude toward health-based applications*:

This sub-theme revealed the participants' attitude toward health-related applications, especially the ArogyaSetu app, which was an Indian Government initiative to track and alert people with COVID-19 symptoms. Most of the participants had a positive attitude toward the initiative. For example, a female respondent, SSD, opined that “*Although the purpose of developing the ArogyaSetu app was good. But I feel it must have incorporated a few more amenities in it. For instance, it shows the number of ill or COVID-19 positive patients around us in terms of the radius (kilometers). Instead, it could be developed to inform us about their street and can give some alarming sound when we are around 100-200 meters closer to that area*.” Although overall response indicated a positive attitude, some acknowledged that they had not installed any other health-related apps as they were not of much use and *ArogyaSetu* was installed only because it was mandatory.

Studies indicated similar findings that emphasize the user's acceptance of the ArogyaSetu app, however, users have also suggested the possible inclusive modifications that would help the people, in general, to understand the details of containment zones, tracking systems, and to some extent prevent the transmission of fake news (Kodali et al., 2020).

### Traditional Indigenous Practices

The techno experts' ambiguous attitude toward the standard protocols have led them to incorporate their traditional and cultural knowledge and practices with scientific knowledge.

*i) Inclination toward Indigenous Knowledge*

A very important theme emerged with an emphasis on knowledge, which indicates the inclination to trend toward age-old customs with a scientific realm. While the study has participants who represent technology experts with a strong scientific background, their Indian roots seemed to be highlighted when science has not been able to provide a vaccine for COVID-19. For instance, a male respondent, AS, emphasized that “*In the end, our culture comes back to us. Traditionally we were following all these things for being healthy and boosting immunity, which then stopped. And now in this uncertain situation, we are shifting again to these traditional practices*.”

This theme helps in understanding the Indian cultural context and the influence it has on individuals, irrespective of the professional background. The responses indicated that, when in dilemma, individuals–including the ones who practice science–might eventually resort to traditional practices. However, it does not mean a total abolition of science but an integration of science with traditional practices. These also indicate toward Indian conformity values. Society and individuals are interdependent and interrelated. People welcome change but those which are more acceptable are incremental. This would ensure a more perfect balance between the new and old ideas which could be more acceptable (Dev and Babu, 2007). The integration of traditional and modern science and exploring it with randomized clinical trials might eventually help in combating future viruses also (Kumar, 2020; Pathania et al., 2020).

*ii) Measures for Immunity Boosting*

This sub-theme emphasized the various preventive measures taken up by people and their attitude toward it to boost immunity. Some health-related news suggested that sources like sunlight, because of vitamin D, vitamin C, Zinc tablets, and ayurvedic *Kada*, which refers to traditional herb-based drinks, help in immunity boosting. Participants had a mixed response toward this.

A male subject, PCS, said that “*Vitamin D shows [an] effect against respiratory disease and antiviral effect but there is need for substantial evidence for its therapeutic use for COVID-19*.” Another male respondent, JP, said, “*Vitamin C and zinc tablets are responsible for boosting or triggering specific immune powers which would help combat viral infection*.” Yet another female subject, SS, said that “*Ayurvedic Kada helps to fight colds and good for immunity as it contains a lot of medicinal based herbs*.”

To prevent infections or viral diseases, people did resort to consuming these preventive tablets even though they are not aware or believe they are not properly proven by scientific research studies. A study indicated that, though traditional, many indigenously-based herb drinks or fruits contain medicinal properties that have been exclusively used and practiced by the ancestors in India (Ganguly, 2013). Older generations are highly respected, and their wisdom and experience are highly regarded which is why their methods are being followed and passed on to generations in India (Krishnan and Mahadevan, 1992). This study's findings also confirm the still persistent and importantly influential Indian values.

## Conclusion

The findings revealed that techno trends awareness of techno experts aided in maintaining social connections during the pandemic. People connected with family and friends through digital means; although the interactions did not meet their physical needs, it still helped them to obtain a feeling of belongingness and social support during home quarantine, thereby reducing the feelings of loneliness, boredom, and other negative effects. An important finding emphasizes online reunions with long lost friends and acquaintances. The techno experts opined that, due to a lack of health facilities, home quarantine is the best choice for asymptomatic patients. Also, they emphasized that, although RT-PCR tests are more reliable and accurate, the current conditions required diagnosis using the rapid antigen tests which are not as accurate but fast. Findings also show that the techno experts' and technical professionals' cultural and professional identity influenced them to integrate science and tradition amid a lack of relevant scientific research and ambiguity in the standard protocols for COVID-19. The study also found that a few experts shifted to traditional healing practices to boost their immunity. Technological advancements have made life easier in many ways, especially in diagnosing COVID-19, in reducing the spread of COVID-19 by using telemedicine services, and in the usage of drones and *ArogyaSetu* apps. Overall, the findings indicated that the experts had good techno trend awareness to maintain social connections and mitigate the impact of the COVID-19 pandemic. Besides, the findings also showed the experts' opinion that if people take responsibility for their health then they would follow the protocols and thus help in mitigating the factors for the spread of COVID-19.

## Implications

The study emphasizes the techno experts' awareness and attitudes toward the recent technology aids that were used to diagnose and mitigate COVID-19. Since they represent individuals who are directly in contact with COVID-19 patients and directly engaged in the diagnostic, treatment, and preventive protocols, this study explores their level of acceptance and attitude toward these facilities. This research paves a way toward understanding the role of integrating different medicinal approaches for a common treatment, suggesting the importance of having an eclectic and multidimensional approach. COVID-19 in India particularly has ignited and aided individuals to reconnect with their traditional roots, which have greater potential for future research. Further, research in exploring these traditional practices would help in revisiting and inheriting the almost forgotten indigenous practices and norms. The current study also highlights the importance of social connectedness and belongingness through digital interactions to maintain relationships and social bondage. Irrespective of the mode of interaction, social relationships are of higher importance especially at a time where it is greatly needed. These perceptions indicate toward the underlying Indian culture values which still stay strong.

## Limitations and Recommendations

Up against the plans of the study, researchers, amidst adversities of the pandemic situation, could not obtain a larger representation of the population. Also, owing to the surge in new cases and increased testing rates, the techno experts with their busy schedule of testing and allied responsibilities could not allocate sufficient time to help researchers provide in-depth data. Further research could be directed toward the differences in the attitude, awareness, and perception of technology trends and social connectedness between the techno experts and other individuals. The present findings contribute to improving the quality of health-related and other technological applications and software which would enhance individuals' quality of life. The study also emphasizes the feasibility and practicability of certain applications that would promote the design of better functioning, accurate, and user-friendly apps in future. Further, research with a more diverse sample concerning the individuals' cultural perspective and moral values would help to understand the deep-rooted beliefs that might play a role in technology acceptance.

## Data Availability Statement

The raw data supporting the conclusions of this article will be made available by the authors, without undue reservation.

## Ethics Statement

The studies involving human participants were reviewed and approved by Departmental Review Board, Central University of Karnataka. The patients/participants provided their written informed consent to participate in this study.

## Author Contributions

All the authors contributed equally during all the stages of this research work. Each author has given their full consent and approval for finalizing the schedule, data collection, analysis as well as submission of the manuscript. To understand the technical information/jargon given by the respondents, a special contribution has been made by NM.

## Conflict of Interest

The authors declare that the research was conducted in the absence of any commercial or financial relationships that could be construed as a potential conflict of interest.
