# Supplementary figures and images for: Techno Trend Awareness and Its Attitude Towards Social Connectedness and Mitigating Factors of COVID-19
Source: Front Psychol. 2021 May 25;12:637395. doi: 10.3389/fpsyg.2021.637395 (PMC8185047; doi:10.3389/fpsyg.2021.637395)

# CONCEPTUAL FRAMEWORK

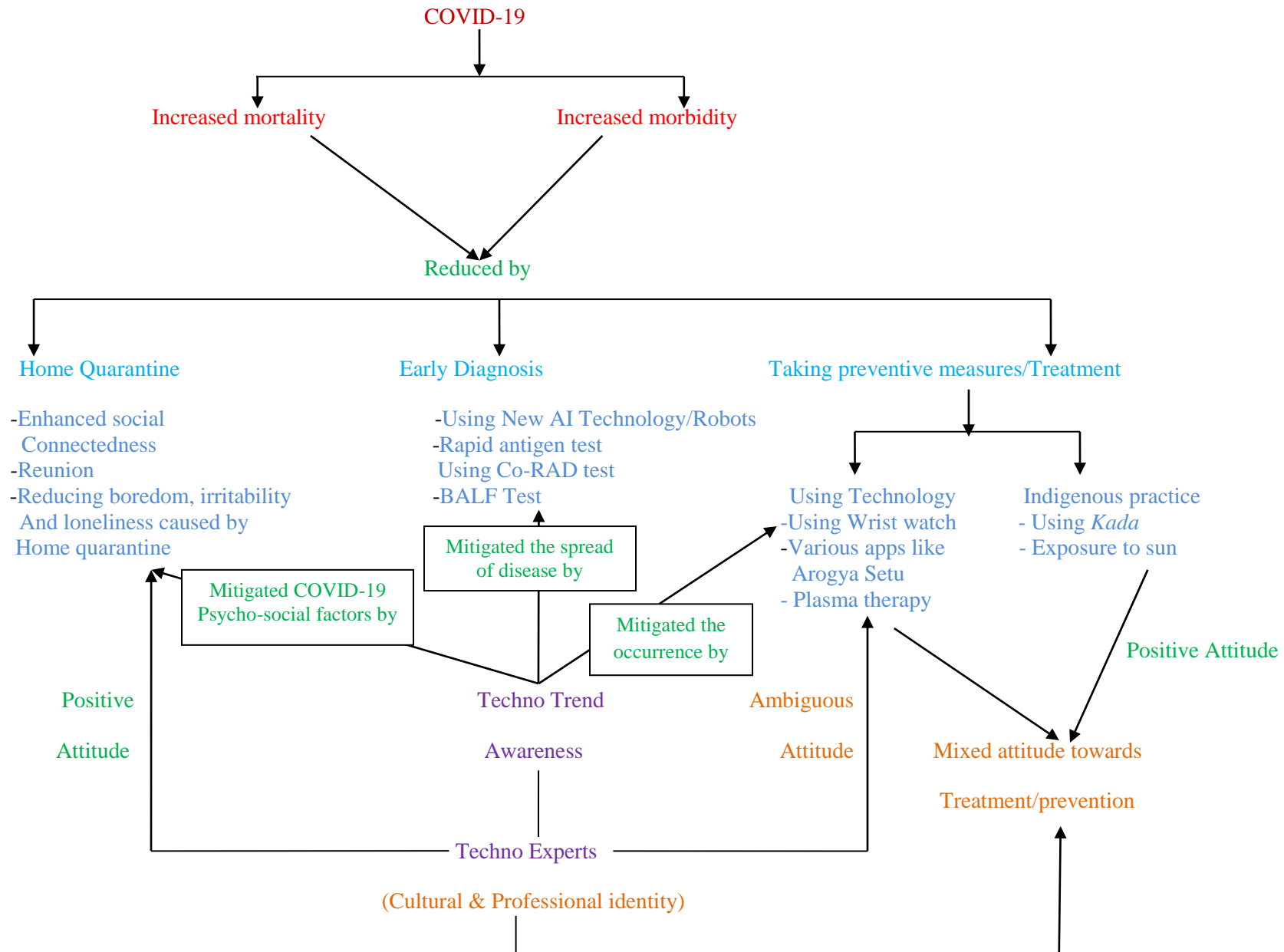

(Source: Prepared by Authors)

Supplement: Supplementary file 1 [file Data_Sheet_1.PDF]
