# Supplementary material for: Techno Trend Awareness and Its Attitude Towards Social Connectedness and Mitigating Factors of COVID-19
Source: Front Psychol. 2021 May 25;12:637395. doi: 10.3389/fpsyg.2021.637395 (PMC8185047; doi:10.3389/fpsyg.2021.637395)
Supplement: Supplementary file 2 [file Data_Sheet_2.PDF]

Themes and Sub-themes extracted from the responses of the Techno Experts

THEMES	SUB-THEMES
Home Quarantine and Social Connectedness: Perception, awareness and attitude	Perception and Attitude towards Home Quarantine
	Attitude towards online interaction
	Online Social Reunion
Early Diagnosis: Awareness and Attitude towards COVID-19 diagnostic tests	Rapid and Non-rapid Testing
	Awareness and Attitude towards BALF test
	Awareness and Attitude towards Physiological measures
	CO-RAD Efficiency
Treatment and Preventive Measures: Mixed Approach	Techno trends awareness for treatment
	Techno trends awareness for prevention
	Traditional Indigenous Practices

(Source: Prepared by Authors)
